# Low rates of endothelial cell dysfunction and transplant‐related mortality in 537 children receiving fludarabine–treosulfan conditioning for all transplant indications: A retrospective multicentre study on behalf of the UK Paediatric BMT group

**DOI:** 10.1111/bjh.20184

**Published:** 2025-06-01

**Authors:** Thomas Altmann, Kanchan Rao, Ramya Hanasoge‐Nataraj, Katharine Patrick, Ben Carpenter, Rachael Hough, Anna‐Maria Ewins, Sanjay Tewari, Oana Mirci‐Danicar, Mary Slatter, Robert Wynn, Su Han Lum

**Affiliations:** ^1^ Translational and Clinical Research Institute Newcastle University Newcastle upon Tyne UK; ^2^ Paediatric Immunology Great North Children's Hospital Newcastle upon Tyne UK; ^3^ Bone Marrow Transplantation, Level 4, CBL Great Ormond Street Hospital London UK; ^4^ Blood and Marrow Transplant Unit Royal Manchester Children's Hospital Manchester UK; ^5^ Department of Paediatric Haematology Sheffield Children's Hospital Sheffield UK; ^6^ Adolescent Haematology University College London Hospitals NHS Foundation Trust London UK; ^7^ Department of Haematology Royal Hospital for Sick Children Glasgow UK; ^8^ Paediatric Haematology King's College London School of Medicine, King's College Hospital London UK; ^9^ Bone Marrow Transplant Unit Bristol Royal Children's Hospital Bristol UK; ^10^ Central Manchester University Hospital Manchester UK; ^11^ Blood and Marrow Transplant Manchester UK; ^12^ Department of Paediatric Haematopoietic Stem Cell Transplant, New Victoria Wing Great North Children's Hospital Newcastle Upon Tyne UK

**Keywords:** children, stem cell transplantation, treosulfan

To the Editor,

The approach to conditioning for allogeneic haematopoietic stem cell transplantation (HSCT) has evolved from toxic myeloablative conditioning to reduced toxicity conditioning (RTC) to reduce acute toxicities and late effects. Alternative RTC regimens have been used in all indications of HSCT including treosulfan, melphalan and pharmacokinetic‐guided reduced toxicity busulfan. Treosulfan was initially used in combination with cyclophosphamide and other agents to mimic the conventional busulfan–cyclophosphamide before its use changed to a combination with fludarabine +/− thiotepa. This multicentre retrospective study reported first HSCT outcomes in 537 children and adolescents after treosulfan–fludarabine (TreoFlu) conditioning for all HSCT indications within the United Kingdom.

All patients who were under 18 years of age at HSCT and received their first HSCT using treosulfan‐based conditioning were identified from nine paediatric transplant centres in the United Kingdom. Five hundred and forty‐eight patients received treosulfan‐based conditioning between 2015 and 2021; 11 were excluded from the study because treosulfan was not used in combination with fludarabine (2 treosulfan only; 2 treo‐thiotepa; 2 treosulfan–cyclophosphamide; 5 treosulfan–cyclophosphamide–melphalan). A questionnaire was sent to the participating centres to obtain data for the study analyses. Primary outcomes were transplant‐related mortality (TRM) and cumulative incidence (CNI) of endothelial cell dysfunction (ECD). ECD included patients who developed veno‐occlusive disease (VOD) and/or transplant‐associated microangiopathy (TMA). Other outcomes accessed were overall survival (OS), graft‐versus‐host disease (GvHD), graft failure, relapse and chimerism. Subgroup differences in OS and TRM were evaluated via the log‐rank test. Competing risks methods were used for the CNIs of ECD and GvHD with competing events death and/or graft failure, and subgroup differences were evaluated by Gray's test.

Patient and HSCT characteristics are summarized in Table 1. Five hundred and thirty‐seven patients received TreoFlu during the study period. The median age at HSCT was 3.9 years (range: 0.1–19). The indications for HSCT included acute lymphoblastic leukaemia (ALL, *n* = 39, 7.3%), acute myeloid leukaemia (AML, *n* = 71, 13.2%), haemoglobinopathy (*n* = 53, 9.9%), severe combined immunodeficiency (SCID, *n* = 42, 7.8%), non‐SCID inborn errors of immunity (non‐SCID IEI, *n* = 268, 49.9%) and metabolic diseases (*n* = 11, 2%). Donors were matched family donor (MFD, *n* = 169, 32%), matched unrelated donor (MUD, *n* = 269, 50%), mismatched family/unrelated donor (MMFD/MMUD, *n* = 29, 5%) and haploidentical donor (HID, *n* = 70, 13%). Stem cell sources were marrow (*n* = 215, 40%), unmanipulated PBSC (*n* = 205, 38%), T Cell Receptor (TCR) αβ/CD19 depleted peripheral blood stem cells (PBSC) (*n* = 83, 15%) and cord blood (CB, *n* = 34, 6%). Additional thiotepa was given in 352 (65.5%) patients (malignant, *n* = 123/132, 93.2%; non‐malignant, *n* = 229/405, 56.6%). Serotherapy was used in 90% (*n* = 485) patients; 321 (60%) had alemtuzumab and 164 (30%) had antithymocyte globulin (ATG). 52 (10%) did not receive serotherapy, 34 MFD (marrow, *n* = 33; PBSC, *n* = 1), 10 matched CB and 8 mismatched CB.

**TABLE 1:** Patient and transplantation characteristics.

Patient characteristics	Results	Missing data
Age at transplant, years, median (range)	3.9 (0.1–19)	0
Transplant indication		
Malignant disorders, *n* (%)	132 (25)	0
ALL	39	
AML	71	
Biphenotypic leukaemia	2	
JMML	7	
MDS	36	
Lymphoma	8	
Non‐malignant disorders, *n* (%)	405 (75)	0
Thalassaemia major	32	
Sickle cell anaemia	21	
Diamond Blackfan anaemia	11	
Bone marrow failure	6	
CAMT	4	
Glanzmann's thrombasthenia	3	
Bernard Soulier syndrome	1	
Hereditary spherocytosis	1	
Osteopetrosis	5	
Inborn errors of metabolism	11	
SCID	42	
Non‐SCID IEI	268	
Donor characteristics		
Type of donor, *n* (%)		0
Matched family donor	169 (32)	
Matched unrelated donor	269 (50)	
Mismatched family/unrelated donor	29 (5)	
Haploidentical donor (**≥**2 antigen MM)	70 (13)	
Stem cell source, *n* (%)		
Marrow	215 (40)	0
Unmanipulated PBSC	205 (38)	
TCRαβ/CD19 depleted PBSC	83 (15)	
CB	34 (6)	
Graft details		
TNC, ×10^8^/kg, median (range)	8.0 (0.37–98.8)	0
CD34, ×10^6^/kg, median (range)	8.4 (0.09–60.9)	
CD3, ×10^8^/kg	2.5 (0.02–283)	
Conditioning, *n* (%)		
Fludarabine–treosulfan–thiotepa	352 (66)	0
Treosulfan–fludarabine	185 (34)	
Serotherapy, *n* (%)		
None	52 (10)	0
ATG grafalon	70 (13)	
ATG thymoglobulin	94 (17)	
Alemtuzumab	321 (60)	
GvHD prophylaxis, *n* (%)		
None	42 (8)	0
CSA alone	158 (29)	
CSA + MMF	290 (54)	
CSA + MTX	36 (7)	
Others	11 (2)	
Transplant outcomes		
Neutrophil engraftment		
Number of patients achieving neutrophil engraftment, *n* (%)	526 (98.0)	0
Days to neutrophil recovery, median (range)	16 (7–73)	
Platelet engraftment^a^		
Number of patients achieving platelet engraftment, *n* (%)	480 (93.6)	12
Days to platelet recovery, median (range)	16 (2–82)	

Abbreviations: ALL, acute lymphoblastic leukaemia; AML, acute myeloid leukaemia; ATG, antithymocyte globulin; CAMT, congenital amegakaryocytic thrombocytopenia; CSA, Cyclosporine A; GvHD, graft‐versus‐host disease; JMML, Juvenile myelomonocytic leukaemia; MTX, methotrexate; MDS, myelodysplastic syndrome; MMF, Mycophenolate Mofetil; non‐SCID IEI, non‐SCID inborn errors of immunity; SCID, severe combined immunodeficiency; TNC, Total Nucleated Cell (dose).

^a^ Platelets did not drop <20 × 10^9^/L in 12 patients; five died before platelet engraftment.

Neutrophil engraftment was achieved in 526 (98.0%) of patients with a median day to neutrophil engraftment of 16 days (range, 7–73). Nine (1.7%) had primary aplastic graft failure (2 MFD, 5 MUD, 1 MMUD, 2 HID) and 2 (0.3%) patients died before achieving neutrophil engraftment. Platelet engraftment was achieved in 93.6% with a median day to platelet engraftment of 16 days (range, 2–82 days).

One‐year CNI of ECD was 5.9% (4.1%–8.4%) (Figure 1A). CNI of VOD and TMA were 2.1% (1.2%–3.8%) and 3.8% (2.4%–5.8%) respectively. Additional thiotepa was not associated with increased CNI of VOD (TreoFlu, 0.5%, 0%–3.9% vs. TreoFlu‐Thiotepa, 2.9%, 1.6%–5.4%, subhazard ratio (SHR) 1.98, 95% confidence interval (CI) 0.56–6.97, *p* = 0.27) and TMA (TreoFlu 3.3%, 1.5%–7.4% vs. TreoFlu‐Thiotepa, 4.1%, 2.4%–7.0%, SHR 1.00, 0.40–2.52, *p* = 0.99). Day‐100 CNI of grade II–IV and grade III–IV acute GvHD (aGvHD) were 27% (22%–32%) and 6% (4%–9%) respectively. Additional thiotepa was not associated with an increased risk of grade II–IV aGvHD (TreoFlu, 28%, (range 21%–37%) vs. TreoFlu‐thiotepa, 36%, (range 21%–33%), SHR 0.92, range 0.65–1.30, *p* = 0.65), or grade III–IV aGvHD (TreoFlu, 7%, (range 4%–13%) vs. TreoFlu‐Thiotpea, 5%, range (3%–9%), SHR 0.79, (range 0.38–1.65). *p* = 0.54). One‐year CNI of chronic GvHD (cGvHD) was 6% (3%–8%).

**FIGURE 1:**
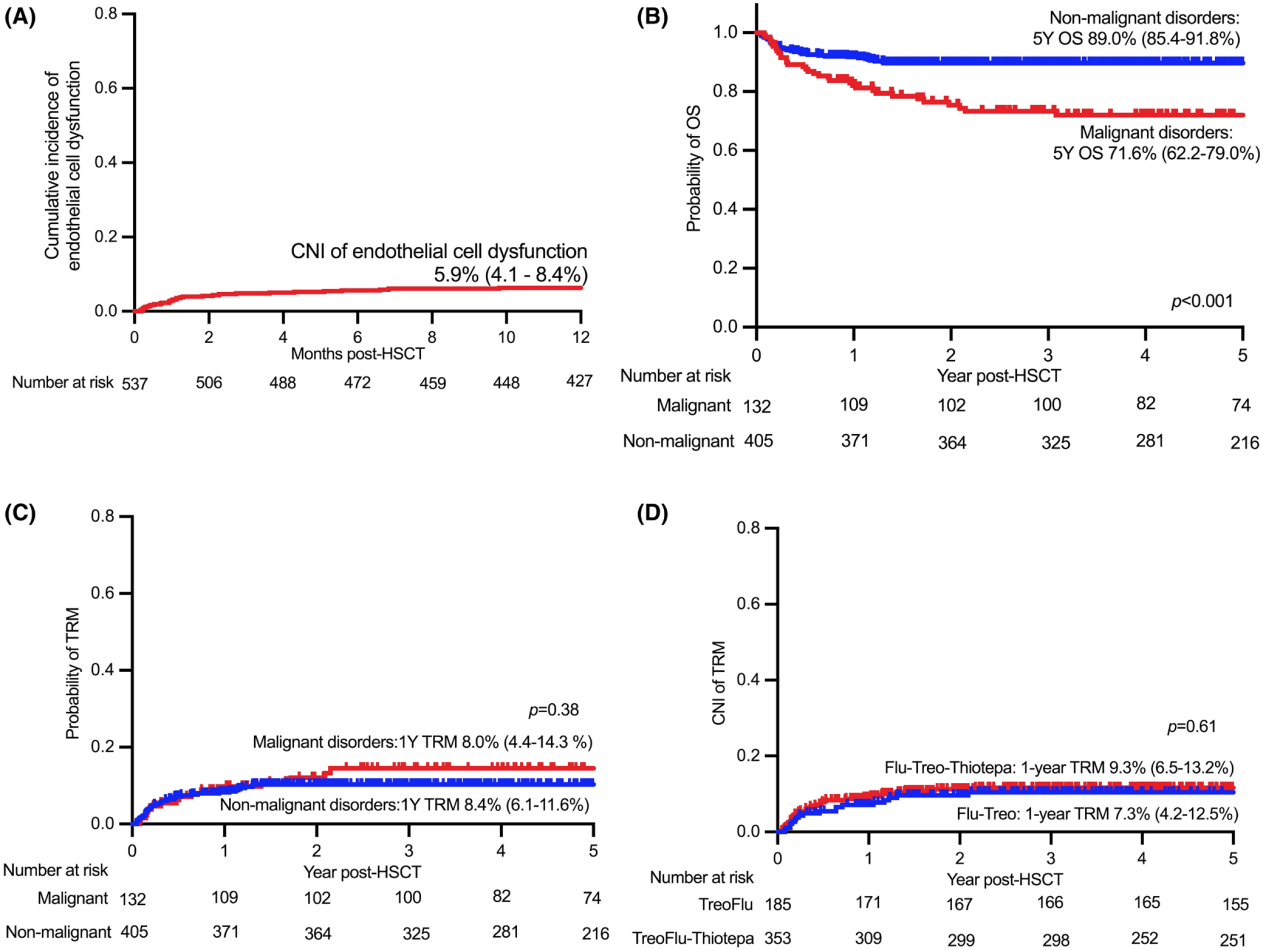
(A) Cumulative incidence of endothelial cell dysfunction; (B) Overall survival of malignant and non‐malignant patients; (C) probability of TRM; (D) cumulative incidence of TRM. TRM, transplant‐related mortality.

The median follow‐up for surviving patients was 6.5 years (range, 3.1–10.0 years). The 5‐year OS was 84.6% (95% confidence interval (CI), 81.0%–87.6%) for the entire cohort; 89.0% (85.4%–91.8%) for non‐malignant disorders and 71.6% (62.2%–79.0%) for malignant disorders (*p* < 0.001, Figure 1B). CNI of TRM was 8.0% (4.4%–14.3%; SHR 1.17, 0.65–2.10, *p* = 0.38) for malignant and 8.4% (6.1%–11.6%) for non‐malignant disorders (Figure 1C). Additional thiotepa was not associated with increased TRM (TreoFlu 7.3%, 4.2%–12.5% vs. TreoFlu‐Thiotepa 9.3%, 6.5%–13.2%, SHR 1.14, 0.65–1.99, *p* = 0.64; Figure 1D). Fifty‐six died of TRM; the causes were infection (*n* = 23), multiorgan failure (*n* = 10), respiratory failure (*n* = 9), encephalopathy (*n* = 3), TMA (*n* = 2), VOD (*n* = 1), GvHD (*n* = 1), others (*n* = 3) and unknown (*n* = 4).

Forty‐four (8.2%) patients received second procedures: 30 s conditioned transplants (14 for graft failure, 1 refractory acute GvHD and 15 for malignancy relapse). In the malignant cohort (*n* = 132), CNI of relapse was 22.8% (16%–35%) at 1 year and 33.5% at 3 years. Latest donor chimerism data were available for 412 of 423 patients who were alive and engrafted after first transplant. The median interval between transplant and latest chimerism result was 6.7 years (range: 3.1–10.0 years). Full myeloid (≥90%) was observed in 331 patients (80.3%), 50 (12.1%) had mixed myeloid (20%–89%) chimerism and 31 (7.5%) had low myeloid (<20%) chimerism. T‐lymphocyte chimerism was full in 362 (87.9%), mixed in 31 (7.5%) and low in 7 (1.6%).

This study provides a snapshot of the experience of using treosulfan‐based conditioning in the United Kingdom. Our study demonstrates that TreoFlu conditioning has been associated with low rates of TRM and ECD in all indications for transplant. TRM for both malignant and non‐malignant cohorts is less than 10%. The incidence of VOD was very low at 2.1% compared to busulfan, which was associated with a high VOD rate of up to 20%–30%, despite pharmacokinetic guided dosing being used.^1^ Additional thiotepa was not associated with increased rates of VOD or TMA. Low rates of severe acute GvHD and chronic GvHD were observed in the entire cohort, of whom 90% received serotherapy.

Comparison between treosulfan–fludarabine and busulfan–fludarabine has been a constant discussion in the transplant community and has been evaluated in numerous studies in children. A phase II multi‐centre randomized controlled study comparing busulfan–fludarabine and treosulfan–fludarabine in non‐malignant diseases was published recently.^2^ This study demonstrated treosulfan was associated with a clinical trend towards reduced TRM at 100 days and 1 year after transplant. Despite an equal proportion of patients receiving additional thiotepa in both groups, increased mixed donor chimerism, higher rates of secondary graft failure and second procedures were observed in the treosulfan group. Retrospective studies have also shown that treosulfan was associated with a higher incidence of graft failure and mixed donor chimerism in a number of conditions.^3^, ^4^, ^5^ There was only one retrospective study comparing treosulfan‐based (*n* = 11) versus busulfan‐based (*n* = 51) in a small cohort of children with malignancy, which demonstrated comparable TRM, disease‐free survival and OS.^6^ A comparison has been reviewed systematically in adults with AML and myelodysplastic syndrome, which showed that treosulfan was superior in terms of OS, event free survival and TRM with comparable incidences of relapse, aGvHD and cGvHD.^7^

While busulfan exposure has been shown to be associated with transplant outcomes, the role of pharmacokinetic studies (pK) in treosulfan has been explored in a small number of studies. The need for pK remains inconclusive due to variable and inconsistent results from these studies.^8^, ^9^, ^10^, ^11^ Chiesa et al. demonstrated high exposure was associated with an increased risk of mortality while low exposure was associated with poor myeloid chimerism in children with IEI.^8^ Van Der Stoep et al. reported that treosulfan exposure was associated with increased toxicity but found no relationship between treosulfan exposure and chimerism, GvHD, OS and TRM in malignant/non‐malignant diseases.^8^ Considering the higher rate of graft failure and mixed donor chimerism with treosulfan, the role of pK‐guided treosulfan dosing needs further evaluation in larger cohorts.

The present study has several limitations. The dosing practice of using age‐based or body surface area varies between transplant centres. The impact of thiotepa on long‐term graft function has not been extensively evaluated in this analysis. Further studies are in progress on the impact of dosing and addition of thiotepa according to disease‐specific cohorts. While this study demonstrated promising safety and efficacy, it did not examine toxicities such as skin toxicity as these data were difficult to retrieve in a retrospective study. Cutaneous toxicities have not been shown to have long‐term consequences and additional thiotepa does not seem to significantly increase the risk of skin toxicity.^12^ A longitudinal study is required to look at late effects including growth and fertility after receiving treosulfan‐based conditioning. Van der Stoep et al. recently reported a significantly lower incidence of chemical gonadal dysfunction following treosulfan compared to busulfan.^13^

Our study provides additional support for promising results of treosulfan‐based conditioning in allogenic transplant in children. We believe that pK studies could further optimize graft outcomes after TreoFlu conditioning. PanPK studies of all the agents used in conditioning may lead to a personalized approach. A prospective pharmacokinetic study including fludarabine, treosulfan, thiotepa and serotherapy agents has been initiated alongside the Haplo+4Kids trial (ISRCTN11859866) in the United Kingdom.

## AUTHOR CONTRIBUTIONS

T.A. collected the data, interpreted the data and prepared the manuscript. M.S. collected the data, contributed to conceptualizing the research and reviewed the manuscript. R.W., K.R., O.M.‐D., K.P., R.H.‐N., A.M.E., B.C. and S.T. collected the data and reviewed the manuscript. S.H.L. conceptualized the research, collected the data, performed the statistical analysis, interpreted the data and critically reviewed the manuscript.

## CONFLICT OF INTEREST STATEMENT

There are no conflicts of interest to report.

## FUNDING INFORMATION

The authors have no funding or financial disclosures.

## ETHICS STATEMENT

Not applicable to this study.

## PATIENT CONSENT STATEMENT

Patients and/or their parents/legal guardians were consented for the inclusion of their anonymous data for the audit of clinical outcome at the time of transplant consent according to institutional guidelines.

## Data Availability

The data that support the findings of this study are available from the corresponding author.

## References

[1] Bognar T , Bartelink IH , Egberts TCG , Rademaker CMA , Versluys AB , Slatter MA , et al. Association between the magnitude of intravenous busulfan exposure and development of hepatic veno‐occlusive disease in children and young adults undergoing Myeloablative allogeneic hematopoietic cell transplantation. Transplant Cell Ther. 2022;28(4):196–202. 35065280 10.1016/j.jtct.2022.01.013

[2] Sykora KW , Beier R , Schulz A , Cesaro S , Greil J , Gozdzik J , et al. Treosulfan vs busulfan conditioning for allogeneic BMT in children with nonmalignant disease: a randomized phase 2 trial. Bone Marrow Transplant. 2024;59(1):107–116. 37925531 10.1038/s41409-023-02135-9 PMC10781637

[3] Albert MH , Slatter MA , Gennery AR , Gungor T , Bakunina K , Markovitch B , et al. Hematopoietic stem cell transplantation for Wiskott‐Aldrich syndrome: an EBMT inborn errors working party analysis. Blood. 2022;139(13):2066–2079. 35100336 10.1182/blood.2021014687

[4] Chiesa R , Wang J , Blok HJ , Hazelaar S , Neven B , Moshous D , et al. Hematopoietic cell transplantation in chronic granulomatous disease: a study of 712 children and adults. Blood. 2020;136(10):1201–1211. 32614953 10.1182/blood.2020005590

[5] Luftinger R , Zubarovskaya N , Galimard JE , Cseh A , Salzer E , Locatelli F , et al. Busulfan‐fludarabine‐ or treosulfan‐fludarabine‐based myeloablative conditioning for children with thalassemia major. Ann Hematol. 2022;101(3):655–665. 34999929 10.1007/s00277-021-04732-4

[6] Olivas‐Mazon R , Bueno D , Sisinni L , Mozo Y , Casado‐Abad G , Martinez AP . A retrospective study of treosulfan versus busulfan‐based conditioning in pediatric patients. Eur J Haematol. 2022;109(5):474–482. 35810360 10.1111/ejh.13828

[7] Zhu S , Liu G , Liu J , Chen Q , Wang Z . Long‐term outcomes of Treosulfan‐ vs. Busulfan‐based conditioning regimen for patients with myelodysplastic syndrome and acute myeloid leukemia before hematopoietic cell transplantation: a systematic review and meta‐analysis. Front Oncol. 2020;10:591363. 33425740 10.3389/fonc.2020.591363 PMC7793760

[8] Chiesa R , Standing JF , Winter R , Nademi Z , Chu J , Pinner D , et al. Proposed therapeutic range of treosulfan in reduced toxicity pediatric allogeneic hematopoietic stem cell transplant conditioning: results from a prospective trial. Clin Pharmacol Ther. 2020;108(2):264–273. 31701524 10.1002/cpt.1715 PMC7484914

[9] van der Stoep M , Bertaina A , Ten Brink MH , Bredius RG , Smiers FJ , Wanders DCM , et al. High interpatient variability of treosulfan exposure is associated with early toxicity in paediatric HSCT: a prospective multicentre study. Br J Haematol. 2017;179(5):772–780. 29048102 10.1111/bjh.14960

[10] Danielak D , Twardosz J , Kasprzyk A , Wachowiak J , Kalwak K , Glowka F . Population pharmacokinetics of treosulfan and development of a limited sampling strategy in children prior to hematopoietic stem cell transplantation. Eur J Clin Pharmacol. 2018;74(1):79–89. 28975382 10.1007/s00228-017-2344-x PMC5748442

[11] Kalwak K , Mielcarek M , Patrick K , Styczynski J , Bader P , Corbacioglu S , et al. Treosulfan‐fludarabine‐thiotepa‐based conditioning treatment before allogeneic hematopoietic stem cell transplantation for pediatric patients with hematological malignancies. Bone Marrow Transplant. 2020;55(10):1996–2007. 32203268 10.1038/s41409-020-0869-6 PMC7515850

[12] van der Stoep M , Bertaina A , Moes D , Algeri M , Bredius RGM , Smiers FJW , et al. Impact of treosulfan exposure on early and long‐term clinical outcomes in pediatric allogeneic hematopoietic stem cell transplantation recipients: a prospective multicenter study. Transplant Cell Ther. 2022;28(2):e91–e99.e97. 10.1016/j.jtct.2021.09.018 34607071

[13] van der Stoep M , Bense JE , de Kloet LC , von Asmuth EGJ , de Pagter APJ , Hannema SE , et al. Effect of Busulfan and Treosulfan on gonadal function after allogeneic stem cell transplantation in children and adolescents with nonmalignant diseases is not exposure‐dependent. Transplant Cell Ther. 2023;29(8):e521–e529.e525. 10.1016/j.jtct.2023.05.003 37156421

